# Effects of Genital Nerve Stimulation Amplitude on Bladder Capacity in Spinal Cord Injured Subjects

**DOI:** 10.1155/2019/1248342

**Published:** 2019-06-24

**Authors:** Shauh-Der Yeh, Bor-Shing Lin, Shih-Ching Chen, Chih-Hwa Chen, Kenneth J. Gustafson, Dennis J. Bourbeau, Chellappan Praveen Rajneesh, Chih-Wei Peng

**Affiliations:** ^1^Department of Urology, School of Medicine, College of Medicine, Taipei Medical University, Taipei, Taiwan; ^2^Department of Computer Science and Information Engineering, National Taipei University, New Taipei City, Taiwan; ^3^Department of Physical Medicine and Rehabilitation, School of Medicine, College of Medicine, Taipei Medical University, Taipei, Taiwan; ^4^Department of Physical Medicine and Rehabilitation, Taipei Medical University Hospital, Taipei, Taiwan; ^5^School of Biomedical Engineering, College of Biomedical Engineering, Taipei Medical University, Taipei, Taiwan; ^6^Department of Orthopedics, Taipei Medical University - Shuang Ho Hospital, Taiwan; ^7^School of Medicine, College of Medicine, Taipei Medical University, Taipei, Taiwan; ^8^Louis Stokes Cleveland VAMC, Cleveland, OH, USA; ^9^Cleveland FES Center, Cleveland, OH, USA; ^10^Departments of Biomedical Engineering & Urology, Case Western Reserve University, Cleveland, OH, USA; ^11^Department of Physical Medicine & Rehabilitation, MetroHealth Medical Center, Cleveland, OH, USA; ^12^Graduate Institute of Biomedical Optomechatronics, College of Biomedical Engineering, Taipei Medical University, Taipei, Taiwan

## Abstract

**Background/Purpose:**

Few studies have investigated the effects of changing the amplitude of dorsal genital nerve stimulation (GNS) on the inhibition of neurogenic detrusor overactivity in individuals with spinal cord injury (SCI). The present study determined the acute effects of changes in GNS amplitude on bladder capacity gain in individuals with SCI and neurogenic detrusor overactivity.

**Methods:**

Cystometry was used to assess the effects of continuous GNS on bladder capacity during bladder filling. The cystometric trials were conducted in a randomized sequence of cystometric fills with continuous GNS at stimulation amplitudes ranging from 1 to 4 times of threshold (T) required to elicit the genitoanal reflex.

**Results:**

The bladder capacity increased minimally and maximally by approximately 34% and 77%, respectively, of the baseline bladder capacity at 1.5 T and 3.2 T, respectively. Stimulation amplitude and bladder capacity were significantly correlated (*R* = 0.55,* P* = 0.01).

**Conclusion:**

This study demonstrates a linear correlation between the stimulation amplitude ranging from 1 to 4T and bladder capacity gain in individuals with SCI in acute GNS experiments. However, GNS amplitude out of the range of 1-4T might not be exactly a linear relationship due to subthreshold or saturation factors. Thus, further research is needed to examine this issue. Nevertheless, these results may be critical in laying the groundwork for understanding the effectiveness of acute GNS in the treatment of neurogenic detrusor overactivity.

## 1. Introduction

Neurogenic detrusor overactivity (NDO), a condition associated with spinal cord injury (SCI), is characterized by uninhibited bladder contractions in response to bladder filling; these contractions reduce bladder capacity and urinary continence [1, 2]. Standard interventions, including cauterization strategies, medications, behavioral controls, and surgery, have been used to treat NDO. However, NDO is often refractory to behavioral and physical therapies. Pharmacological treatments such as antimuscarinic medication effectively treat neurogenic bladder; however, they often cause side effects and deteriorate the subjects' quality of life [2, 3]. Electrical stimulation is an alternative therapeutic approach for individuals with SCI and neurogenic bladder.

Various electrical neuromodulation approaches have been developed for NDO treatment. For example, sacral nerve stimulation (SNS) [4] therapy and transcutaneous tibial nerve stimulation (TTNS) [5–7] therapy have been used to treat subjects with SCI and NDO. Although both TTNS and SNS therapies have been reported to significantly improve urinary continence by increasing bladder capacity in subjects with incomplete SCI [5–7], a few recent studies report that TTNS and SNS did not produce a satisfactory outcome in related clinic trials [8, 9]. Moreover, these two neuromodulation approaches are not effective for increasing bladder capacity in individuals with complete SCI; it is still unclear why both approaches failed to improve urinary continence in these individuals. One possible explanation may be attributable to the fact that the therapeutic mechanism of these approaches might require the neuromodulation of supraspinal-mediated neural pathways [10].

Dorsal genital nerve stimulation (GNS) is a promising treatment for NDO; unlike TTNS and SNS therapies, it is applicable to subjects with complete as well as incomplete SCI because the neuromodulation mechanism mainly involves the regional spinal reflex pathways in the lower urinary tract [11]. In addition, GNS for modulating NDO involves surface electrical stimulation that reduces the risk of iatrogenic infections or surgical complications [12], but SNS therapy may require complex implant surgery.

Numerous studies[13–17] have demonstrated that GNS effectively inhibits NDO and improves continence in individuals with NDO. Wheeler et al. [16] conducted GNS in individuals with SCI by using surface electrodes. The treatment significantly increased the bladder filling volume, as assessed using cystometrography, and the increase in bladder capacity was further confirmed through a carry-over benefit of the stimulation [13, 15] that persisted even after stimulation was terminated. Goldman et al.[11] provided GNS treatment for 7 days to women with overactive bladder; the results demonstrated that the treatment alleviated the symptoms of overactive bladder. Furthermore, a recent study demonstrated that individuals with pelvic sensation can tolerate GNS at amplitudes that effectively inhibit NDO [18].

Although many studies have confirmed the effects of GNS on NDO, only a few have investigated the amplitude-dependent effects of GNS on bladder inhibition in individuals with SCI. The present study quantified the effects of acute electrical stimulation of the dorsal genital nerve of the penis on bladder inhibition and cystometric capacity gain in individuals with SCI. The results of this study will improve our understanding of the relative efficacy of stimulation amplitudes and facilitate identification of the optimum stimulation parameters for clinical research and treatment.

## 2. Methods

### 2.1. Subject Recruitment

This study was conducted at Taipei Medical University Hospital and was approved by its institutional review board (N201605025). Subjects with SCI and NDO diagnosed using urodynamics were included. The inclusion criteria were as follows: suprasacral SCI with bladder overactivity; neurological stability, no change in motor or sensory function in the past one month; skeletal maturity, age > 18 years; no ureteric reflux; no urinary tract infection; no onabotulinumtoxinA injection within the past 6 months; and no significant urethral trauma. Subjects with active sepsis, active pressure sores in the pelvic region, and the inability to speak were excluded. All the subjects were enrolled at least 6 months after SCI. If the subjects were actively using anticholinergic medications (to relax the bladder), they were asked to stop them for 2 weeks before the study. Of the 20 subjects screened in an interview, 10 did not meet the inclusion criteria; thus, 10 men were included in this study.

### 2.2. Urodynamic Measurement

A 7-French dual-lumen Foley catheter was inserted into the bladder through the urethra, and the bladder was emptied (volume was recorded). One lumen of the catheter was connected to a pressure transducer and amplifier to record bladder pressure, and the other was used to control bladder volume by filling it with sterile saline and subsequently emptying it. A balloon catheter inserted in the rectum was used to determine the detrusor pressure by subtracting the rectal from vesical pressure. Surface electromyogram electrodes were applied to the anal sphincter to detect the genitoanal reflex and monitor pelvic floor activity. The minimum stimulation amplitude necessary to elicit the genitoanal reflex was defined as the stimulation threshold (T). The reflex stimulation threshold was determined by the onset of a visible or palpable reflexive external anal sphincter contraction. The sensation threshold was also observed during the genitoanal reflex test. The sensation threshold was determined by increasing the amplitude in increments of about 1 mA and retesting until the subject could perceive stimulation. Serial cystometrograms were obtained by filling the bladder with saline (at 30 mL/min) and recording the volume of and pressure in the bladder. Infused volumes were measured using a force transducer and an infusion pump. All signals were monitored using a urodynamic system (Solar Gold, Medical Measurement Systems, Williston, VT, USA).

### 2.3. Genital Nerve Stimulation

Two round surface electrodes (2 cm in diameter; Natus, Middleton, WI, USA) were placed on the dorsum of the penile shaft, 2 cm apart, to provide bilateral GN stimulation. The electrodes were connected to a stimulator (Continuum, Empi, Clear Lake, SD, USA). The minimum (or threshold) stimulation amplitude (T) necessary to elicit the genitoanal reflex was first determined for each subject. Subsequently, each subject received a sequence of 5~6 cystometric fill trials. Subjects first received a control fill without GNS to verify the diagnosis of neurogenic detrusor overactivity and then a series of cystometric fill trials with and without continuous GNS, randomized. The GNS current was continuously applied during a cystometric fill between the start of bladder infusion and the onset of uninhibited bladder contraction of more than 15 cmH_2_O. GNS was applied as a continuous, rectangular biphasic, charge-balanced current (fixed frequency at 20 Hz and pulse width at 200 *μ*s) [19, 20] at stimulation amplitudes of 1, 2, 3, and 4T. The tolerance limit was determined during GNS trials. The largest GNS amplitude was applied during a cystometric fill trial and the subject did not complain of any discomfort. This amplitude was defined as the tolerance limit.

### 2.4. Data Analysis

**Figure 1:**
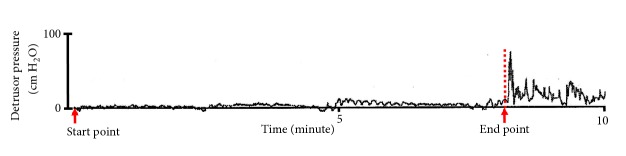
The calculation of bladder compliance. The start point for the compliance calculation was the detrusor pressure at the start of bladder filling and the corresponding bladder volume (usually zero), and the endpoint for compliance calculations was the detrusor pressure (and corresponding bladder volume) immediately before the start of any detrusor contraction that caused significant leakage. Thus, the bladder compliance was calculated as the ratio of the change in bladder volume to the change in detrusor pressure during bladder filling (ΔDV/ΔDP).

The effect of GNS on bladder capacity (primary outcome) and bladder compliance (secondary outcome) was assessed through acute urodynamic measurement. Bladder capacity data from randomly selected control cystometric fills in each subject was used to determine the baseline value, rather than just using the first control cystometrogram for a baseline measurement. The bladder compliance was calculated as the ratio of change in volume infused into the bladder to the change in detrusor pressure during bladder filling (ΔDV/ΔDP). The start point for the calculation of bladder compliance was the detrusor pressure at the start of bladder filling and the corresponding bladder volume, and the endpoint for compliance calculations was the detrusor pressure (and corresponding bladder volume) immediately before the start of any detrusor contraction that caused significant leakage [21], as shown in Figure 1.

All data were presented as means ± standard deviations (SDs). Analysis of variance (ANOVA) was used to compare the effects of different stimulation amplitude changes on bladder capacity and bladder compliance. ANOVA was followed by LSD honest significant difference (HSD) post hoc test of paired comparisons (SigmaStat, SPSS, Chicago, IL). In this study, Pearson correlation and linear regression analyses were used to investigate the association between stimulation amplitude and bladder capacity. Values of* P* < 0.05 were considered statistically significant in all the data analyses.

## 3. Results

### 3.1. Subject Characteristics

In this study, 10 subjects (mean age: 35.6 ± 15 years, range: 22–65 years) were initially recruited for urodynamic measurements. Based on the urodynamic results, two subjects (Nos. 5 and 7) were not included in the final data analysis because they did not receive diagnoses of NDO. The subject characteristics are presented in Table 1.

### 3.2. Sensation Threshold and Tolerance Limit

The injury severity of eight subjects included 5 individuals with incomplete SCI and 3 individuals with complete SCI. All subjects with incomplete SCI could sense stimulation and the average sensation threshold was 7.4 ± 3.0 mA (mean of five incomplete subjects), whereas none of the individuals with complete SCI could sense stimulation (Table 2). The minimum stimulation amplitude (1T) required to elicit the genitoanal reflex was successfully detected in all subjects, where the average stimulation threshold was 12.6 ± 2.7 mA (mean of eight subjects).

The tolerance limit was 3.6 ± 0.5 times the genitoanal reflex. All subjects with incomplete or complete SCI tolerated continuous GNS at 1-3T. When the continuous GNS amplitude was elevated to 4T, all individuals with complete SCI (*n* = 3 out of 3) could tolerate this stimulation intensity. However, three individuals with incomplete SCI (*n* = 3 out of 5) did not tolerate GNS at the 4T amplitude. They (subjects #1, #2, and # 9) reported that they felt an uncomfortable pain or sensation and wanted to stop this intensity level of GNS. Therefore, continuous GNS with 4T amplitude during bladder filling was not examined in these three subjects. GNS was not observed to trigger autonomic dysreflexia in any of the subjects.

### 3.3. Effects of GNS Amplitude on Bladder Capacity

The minimum stimulation amplitude (1T) required to elicit the genitoanal reflex ranged from 5.5 to 15 mA, as shown in Table 1. According to the corresponding threshold values, each subject received a randomized sequence of cystometric fill trials with GNS. The GNS was randomly provided at 1, 2, 3, and 4T. All the subjects exhibited increased bladder volumes with GNS during the cystometric trials.  Table 3 summarizes the minimal and maximal responses of bladder capacity at various GNS amplitudes. The minimal and maximal increases in bladder capacity were 34.4%  ± 22.0% and 77.3%  ± 46.8% of the control value, respectively, with stimulation amplitudes of 1.5 ± 0.5 and 3.2 ± 1.0 times T, respectively (range 1–4T). In addition, Table 4 shows the response of bladder capacity in chronological order with a randomized stimulation intensity in each subject. The bladder capacity did not continuously increase along with the order of repeatedly cystometric fills in all subjects; however, the higher stimulation intensities generally had a large bladder capacity increment.

### 3.4. Correlation between Stimulation Amplitude and Bladder Capacity

**Figure 2:**
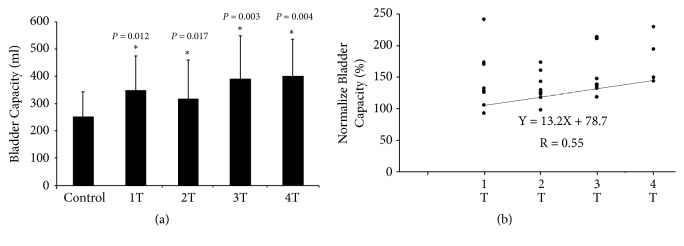
Effects of GNS amplitudes on the response of bladder capacity. (a) The absolute bladder capacities at all tested GNS amplitudes were significantly higher than the control value. Each bar represents the mean ± the standard deviation value. ∗ indicates a statistically significant difference from the control value (*P* < 0.05). (b) A linear correlation (*R* = 0.55) between the changes in stimulation amplitude (X) and bladder capacity (Y) was expressed as Y = 13.2X + 78.7, in which the X ranged between 1T and 4T.

The correlation between the amplitude of GNS and the bladder capacity was further analyzed. Two-factor ANOVA indicated that the absolute bladder capacity was dependent on the stimulation amplitude (*P* = 0.048), but the individual variation (i.e., comparison among eight subjects) did not demonstrate a significant effect on the absolute bladder capacity (*P* = 0.179). These results also indicated that there was no significant difference in bladder capacity gain between subjects with incomplete and complete SCI. Further, post hoc paired comparison indicated that all tested amplitudes (1–4T) significantly increased the bladder capacities to approximately 134%–189% of the control value (*P* < 0.05), as shown in Figure 2(a). High stimulation amplitudes caused larger increases in bladder capacity; thus, stimulation amplitude and bladder capacity were significantly correlated (*R* = 0.55,* F* = 14.366,* P* = 0.01, Figure 2(b)). Furthermore, the linear regression expression relating changes in the bladder capacity (*Y*) and stimulation amplitude (*X*) was as follows.

(1) $$\begin{array}{c} Y=13.2X+78.7\;where\;\;4\geq X\geq 1 \end{array}$$

### 3.5. Bladder Compliance Changes

**Figure 3:**
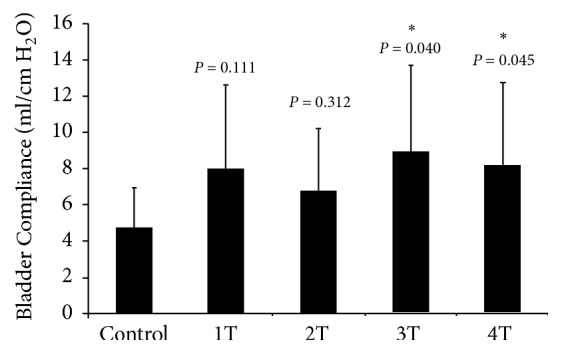
Effects of GNS on bladder compliance. The bladder compliances at 3T and 4T of GNS were significantly increased compared to the control value, but low intensities (1-2T) did not reach a statistical significance. Each bar represents the mean ± the standard deviation value. ∗ indicates a statistically significant difference from the control value (*P* < 0.05).

The effects of GNS on bladder compliance change were further analyzed. One-way ANOVA indicated that the bladder compliance was significantly increased at 3T and 4T of GNS amplitudes (*P* < 0.05, LSD HSD) but 1T and 2T did not demonstrate a significant effect on the bladder compliance (*P* = 0.111 and 0.312, respectively), as shown in Figure 3.

## 4. Discussion

The stimulation amplitude for effective bladder inhibition through surface GNS is typically twice the stimulation threshold for evoking the genitoanal reflex [19]. To our knowledge, only a few studies have reported an amplitude-dependent effect of GNS on bladder inhibition [22]. The present study was the first to analyze the statistical relationship between stimulation amplitude and bladder capacity in the subjects with SCI and NDO.

Our regression analysis showed that the stimulation amplitude was positively linearly correlated with changes in bladder capacity (formula (1)). According to our linear regression results, for every onefold increase in stimulation amplitude (1T), subjects exhibited an approximate increase of 13.2 % in bladder capacity. In practice, usually each subject with SCI underwent a maximum of less than six cystometric fills to ensure that the duration of the experimental session is minimal in order to reduce subject discomfort [15, 18]. Therefore, the tested stimulation amplitudes were selected from a small range of 1-4T and with a large increment of 1T, whereas the stimulation amplitudes higher than 4T were not tested in this study. This small tested amplitude range with a large increment may have influenced the accuracy of the linear regression analysis in the present study. In addition, it has been assumed that a GNS amplitude out of the range of 1-4T may not be a nonlinear model. GNS amplitude below 1T may have no stimulation effect on bladder capacity while the amplitude higher than 4T may not exhibit a linear correlation due to pain sensation or stimulation saturation factors. Hence, additional studies are needed to confirm the accuracy of the relationship between stimulation amplitude and bladder capacity outside of the course range tested here.

The results of this study revealed that bladder capacity was minimally increased, by approximately 34%, when stimulated with an average amplitude of 1.5T. These results were also substantiated by a recent study which demonstrated that GNS can be effective at stimulation amplitudes between 1 and 2 times the genitoanal reflex [18]. However, our results were inconsistent with a study by Previnaire et al. [15]. They concluded that the amplitude of GNS below 2T had no statistically significant effect on the bladder capacity, even though in their study GNS with 1T amplitude substantially enhanced the increase of bladder capacity in six out of ten subjects. This discrepancy may be a result of different stimulation parameters utilized in GNS experiments such as the stimulation frequency, pulse duration, and electrode size.

Although conditional GNS was not performed in the present study, i.e., applied only during bladder contractions, previous studies indicated that both continuous [19] and conditional GNS effectively increased the bladder capacity [23, 24]. Moreover, in these studies, conditional GNS was observed to be more effective in increasing the bladder capacity by at least 8-9% compared to continuous stimulation. This finding may be accounted for by the fact that conditional stimulation could minimize accommodation effects on spinal reflexes due to a shorter stimulation time compared to continuous GNS. However, few studies have compared the effects of GNS amplitude on bladder capacity gain between continuous and conditional stimulation status. According to these studies, it is indirectly speculated that in comparison to continuous GNS, conditional GNS may require a lower stimulation amplitude to achieve an equal gain in bladder capacity. It is also unclear whether there is a difference in the tolerance limit between conditional and continuous GNS in subjects with SCI. Thus, additional research is needed to examine these important issues.

In spite of a positive correlation between the stimulation amplitude and bladder capacity gain, various subject-specific factors also must be considered when determining the maximal amplitude for a GNS clinical trial. For example, the tolerable limit for electrical sensation, stimulation threshold for evoking the genitoanal reflex, and sensation of comfort with respect to electrical stimulation may vary among subjects with SCI. In addition, for the safety of the subjects, the maximal usable GNS amplitude is limited because of various factors, such as the electrical current density over an electrode-tissue interface, variable electrode-tissue impedance, and the onset of electrical burn triggered by an overload of electrical current. Hence, using 2T as the general acceptable stimulation amplitude for effective bladder inhibition in acute GNS trials is reasonable.

In this study, the tested stimulation amplitudes ranged from 5.5 mA to 60 mA and they were smaller than those used in other studies (20–80 mA) [15, 16, 24–27]. This is a possible reason why the onset of autonomic dysreflexia or changes in blood pressure and pulse rate trigged by GNS in any of the subjects were unnoticed during the urodynamic fills. The dorsal genital nerve is a terminal branch of the pudendal nerve. The mechanism underlying the increase in bladder capacity by acute GNS is typically considered to be due to the inhibition of the bladder through pudendal afferent stimulation. The pudendal afferent inputs may reflexively reduce the output of the parasympathetic efferent to the bladder by direct postsynaptic inhibition and possibly by presynaptic inhibition of the bladder afferents [28, 29]. Another potential mechanism is that acute GNS may increase the sympathetic outflow to the bladder (through the hypogastric nerve), which directly inhibits the smooth muscles of the bladder wall [28]. Our results show that GNS produced a significant effect on the bladder compliance gain, which indirectly supports these assumptions. These results show that higher intensities of GNS (3-4T) produced a profound neuromodulation effect on the autonomic nerve system compared to that of low intensity stimulation (1-2T). However, there is a possibility that the excessively high GNS amplitudes may activate pain sensation and result in autonomic dysreflexia, because the cutaneous noxious stimuli may increase spinal sympathetic reflex activity and raise blood pressure below the lesion level of SCI [30]. Nevertheless, the possibility of excessively high GNS amplitudes inducing autonomic dysreflexia warrants further exploration.

Various GNS frequencies have been tested in clinical studies on bladder inhibition in subjects with SCI and NDO, including the use of 5 [15, 16], 10 [31], 15 [24], 20 [18, 20, 25, 27], and 25 Hz [32]. One study indicated that stimulation frequencies of 5, 10, and 20 Hz were equally effective in treating NDO in subjects with SCI [31]. However, the effective GNS frequency did not usually exceed 10 Hz in the animal experiments [33, 34]. Although no consensus has currently been reached regarding the optimal GNS frequency for bladder inhibition, most recent studies have focused on 20 Hz as the optimal stimulation frequency. Thus, a frequency of 20 Hz was used for acute GNS in this study, based on the literature [18, 20, 25, 27].

The present study has several limitations. Our study demonstrates that bladder capacity gain was significantly dependent on the stimulation amplitude. However, due to the limited sample size, we did not further analyze subject-specific factors that may have contributed to the variance in bladder capacity outcome, such as patterns of SCI (incomplete or complete), infusion rates, and anticholinergic medications. Subjects with incomplete SCI and sensory function usually have a lower tolerance for high-amplitude GNS than subjects with complete SCI do [18]. This dissimilarity in the perception of sensation may induce different physiological responses in terms of bladder inhibition in acute GNS experiments.

Most studies have reported a fixed range of infusion rate when obtaining cystometrograms (2–60 mL/min) [19]. According to International Continence Society Good Urodynamic Practices, the normal physiological filling rate is estimated by body weight in kilogram divided by four [35]. Thus, the expected physiological filling rate in the present study should be 15 ml/min (due to 60 kg of average body weight obtained in subjects; Table 1). The actual filling rate in this study was fixed at 30 ml/min in all subjects, which was 2-fold higher than the expected value. The use of a faster infusion rate is a common method of minimizing the length of the experimental session, ensuring subject comfort, and enabling collection of a large amount of data from cystometric measurements. However, a faster infusion rate might strain the compliance of the bladder. In addition, sequential cystometric measurements may increase bladder capacity due the stretching of the detrusor muscle. To avoid these potential influences on bladder capacity outcome, we maintained the infusion rate at 30 mL/min and randomized the cystometric trials, with sufficient intervals to restore equilibrium (at least 15–25 minutes) between trials [18]. Our statistical data showed that the sequential cystometric fills did not produce a dramatic influence on bladder capacity outcome because the stimulation intensity rather than the sequential order of cystometric measurements generally had a large impact on the bladder capacity increment (Table 4). Thus, the increase in bladder capacity should be primarily determined by the stimulation amplitude factor in the present study.

In this study, the subjects used various methods to manage their bladder overactivity condition, including wearing diapers, intermittent catheterization, Foley catheterization, and pharmacotherapy (particularly anticholinergic medications). If the subjects were actively using anticholinergic medications, they were asked to stop taking the drugs 2 weeks prior to the beginning of the study because the medications may mask the effects of GNS. However, we found that most subjects experienced an increase in the frequency of incontinence episodes during the period in which they stopped taking medications. In addition, we observed that the subjects who did not use anticholinergic medications appeared to have smaller baseline bladder capacities and smaller bladder capacity gains after GNS than the subjects who were using anticholinergic medications did. This finding was consistent with those of a previous study [24]. This warrants future explorations of the effects of medication history on acute GNS performance.

Acute GNS effectively inhibited NDO and immediately increased bladder capacity. GNS is not widely applied in clinical practice due to no long-lasting effect compared with antimuscarinics and intravesical onabotulinumtoxinA injection [2, 3]. However, these drugs often cause side effects and deteriorate patients' quality of life; thus, it led many individuals to depend on either indwelling catheters or chronic clean intermittent self-catheterization. Chronic GNS is a potential therapeutic direction to produce a long-lasting effect on bladder inhibition. This is because chronic neuromodulation of the peripheral nerve may effectively alter the central neurosynaptic connections (i.e., neuroplastic effects) [36], compared to a single trial of GNS. One recent systematic review study by Kessler's group compared the efficacy of acute and chronic transcutaneous electrical nerve stimulation (which included GNS) [37]. Their finding suggested that both acute and chronic neuromodulation effectively increased bladder capacity in NDO. Moreover, this chronic approach decreased the number of voids and leakages per day. Nevertheless, as chronic nerve stimulation might produce habituation effects, it remains unclear whether the chronic GNS approach can have a superior clinic outcome on bladder continence function compared to the acute approach. Since few studies have reported on the chronic effects of GNS, there is no assurance that our present results are suitable for application in individuals with SCI during chronic GNS treatment or in a chronic take-home study and it still requires in-depth understanding and detailed research to make that determination.

## 5. Conclusion

This study reveals that acute GNS increases bladder capacity in subjects with SCI and NDO. Bladder capacity and stimulation amplitude (between 1- and 4-fold minimum stimulation amplitude) are significantly linearly correlated. However, the regression relationship between bladder capacity and GNS amplitude over higher intensity (> 4-fold stimulation amplitude) remains unclear; thus, additional research is needed to confirm the applicability of the results. The present study contributes to our understanding of the influence of GNS amplitude in the treatment of neurogenic detrusor overactivity.

## Figures and Tables

**Table 1:** Subject characteristics.

Subject	Age	Body weight	Post injury	SCI	Asia	Bladder	neurogenic
ID	(years)	(kg)	(years)	level	impairment	management	bladder overactivity
1	65	60	1.5	T5	C	FC/D	Yes
2	28	56	1.5	C4	C	FC	Yes
3	23	65	2	T3	B	FC	Yes
4	26	53	5	C3	A	IC	Yes
5	31	68	2	T10	A	IC/VV	No
6	32	73	4	T4	C	IC	Yes
7	54	62	36	L3	C	VV	No
8	26	50	4	T4	A	IC/FC	Yes
9	49	63	5	C4	C	IC/FC	Yes
10	22	50	4.5	C4	A	IC/D	Yes

Average	35.6 ± 14.9	60.0 ± 7.7	6.6 ± 10.4				
	(22 ~ 65 y/o)	(50 ~ 73 kg)	(1.5 ~ 36 years)				

ASIA impairment score is recorded in the standard A through E format. All subjects had upper motor neuron spinal cord injuries. Bladder management strategies include Foley catheterization (FC), diaper (D), intermittent catheterization (IC), and volitional voiding (VV). Subjects 5 and 7 did not receive a diagnosis of neurogenic detrusor overactivity based on urodynamic measurements.

**Table 2:** The sensation threshold, stimulation threshold of genitoanal reflex, and tolerance limit in subjects with SCI.

Subject	Injury	Sensation	Stimulation	Tolerance
ID	severity	threshold	threshold (1T)	limit
1	Incomplete	8	12	36.0 (3T)
2	Incomplete	4	5.5	17.0 (3T)
3	Incomplete	6	14	56.0 (4T)
4	Complete	NA	14	56.0 (4T)
6	Incomplete	12	15	60.0 (4T)
8	Complete	NA	13	52.0 (4T)
9	Incomplete	7	13	39.0 (3T)
10	Complete	NA	14	56.0 (4T)

Average		7.4 ± 3.0 mA	12.6 ± 3.0 mA	46.5 ± 14.7 mA
				(3.6 ± 0.5 T)

**Table 3:** Effects of GNS on bladder capacity in subjects with SCI.

Subjects	Baseline bladder capacity	Min. response		Max. response	
ID		Stimulation amplitude	Bladder capacity gain	Stimulation amplitude	Bladder capacity gain
1	292 mL	1T	73.0%	3T	113.0%
2	391 mL	1T	25.3%	3T	35.6%
3	254 mL	2T	22.8%	4T	43.3%
4	140 mL	2T	60.0%	4T	129.3%
6	169 mL	1T	28.4%	4T	89.4%
8	96 mL	2T	42.7%	1T	140.6%
9	278 mL	2T	17.4%	3T	18.3%
10	402 mL	1T	5.5%	4T	49.3%

Average	253 ± 112 mL	1.5 ± 0.5T	34.4 ± 22.0%	3.2 ± 1.0T	77.3 ± 46.8%

**Table 4:** The response of bladder capacity in chronological order with a randomized stimulation intensity.

Subject	The sequence of cystometric fill trials in each subject				
ID	1st trial	2nd trial	3rd trial	4th trial	5th trial
1	0% (Control)	73.0% (1T) ∗	113.2% (3T)	76.1% (2T)	5.6% (control)
2	0% (Control)	25.8% (2T)	25.3% (1T)	35.6% (3T)	2.6% (control)
3	0% (Control)	43.3% (4T)	22.8% (2T)	31.9% (1T)	31.9% (3T)
4	0% (Control)	70.0% (1T)	112.9% (3T)	60% (2T)	129.3% (4T)
6	0% (Control)	29.6% (2T)	28.4% (1T)	89.4% (4T)	47.3% (3T)
8	0% (Control)	140.6% (1T)	42.7% (2T)	110.4% (3T)	93.8% (4T)
9	0% (Control)	18.0% (2T)	-7.6% (control)	18.3% (3T)	17.4% (1T)
10	0% (Control)	5.5% (1T)	38.6% (3T)	7.4% (2T)	49.3% (4T)

∗ 73.0% (1T) represents a 73.0% bladder capacity gain at 1T stimulation intensity compared to its corresponding control value.

## Data Availability

The clinic datasets used to support the findings of this study have been monitored and deposited in the TMU-eJIRB System of Taipei Medical University (ID: N201605025).
